# Differential effects of conifer and broadleaf litter inputs on soil organic carbon chemical composition through altered soil microbial community composition

**DOI:** 10.1038/srep27097

**Published:** 2016-06-03

**Authors:** Hui Wang, Shi-Rong Liu, Jing-Xin Wang, Zuo-Min Shi, Jia Xu, Pi-Zheng Hong, An-Gang Ming, Hao-Long Yu, Lin Chen, Li-Hua Lu, Dao-Xiong Cai

**Affiliations:** 1 Key Laboratory of Forest Ecology and Environment, China’s State Forestry Administration, Institute of Forest Ecology, Environment and Protection, Chinese Academy of Forestry, No.2 Dongxiaofu, Haidian District, Beijing, 100091, China; 2 Division of Forestry and Natural Resources, West Virginia University, P.O. Box 6215, Morgantown, WV, 26506-6125, USA; 3 Experimental Center of Tropical Forestry, Chinese Academy of Forestry, Pingxiang, Guangxi, 532600, China

## Abstract

A strategic selection of tree species will shift the type and quality of litter input, and subsequently magnitude and composition of the soil organic carbon (SOC) through soil microbial community. We conducted a manipulative experiment in randomized block design with leaf litter inputs of four native subtropical tree species in a *Pinus massoniana* plantation in southern China and found that the chemical composition of SOC did not differ significantly among treatments until after 28 months of the experiment. Contrasting leaf litter inputs had significant impacts on the amounts of total microbial, Gram-positive bacterial, and actinomycic PLFAs, but not on the amounts of total bacterial, Gram-negative bacterial, and fungal PLFAs. There were significant differences in alkyl/O-alkyl C in soils among the leaf litter input treatments, but no apparent differences in the proportions of chemical compositions (alkyl, O-alkyl, aromatic, and carbonyl C) in SOC. Soil alkyl/O-alkyl C was significantly related to the amounts of total microbial, and Gram-positive bacterial PLFAs, but not to the chemical compositions of leaf litter. Our findings suggest that changes in forest leaf litter inputs could result in changes in chemical stability of SOC through the altered microbial community composition.

The composition of plant litter and its rate of input to the forest floor will change due to climate, land-use change, and ecosystem disturbance^1^. A strategic selection of tree species will shift the type and quality of litter inputs, and subsequently the chemical composition of soil organic carbon (SOC) through the altered soil microbial community composition. The changes in soil microorganisms may lead to changes in enzyme activities that can give rise to selective decomposition of some compounds^2^, the metabolic capabilities of decomposers^3^, and/or the chemical composition of soil microbial necromass^4^. The potential for SOC sequestration could accordingly vary with litter type and soil microbial community; however, large uncertainties remain concerning the effects of litter input on soil microbial community composition and the retention of litter-derived C in soils^5^.

Soil contains a mixture of heterogeneous C pools. Through chemical and/or physical methods, bulk soils can be separated into different fractions that differ in chemical composition and/or location in the soil matrix^6^. Soil fractionations based on carbon (C)-rich organic molecules allow bulk soil to be partitioned into functional pools of recalcitrant and labile chemical compositions and can help to assess the degrees of stability against decomposition in the environment^7^,^8^. For example, alkyl C chains in lipids and aromatic structures in aromatics and phenolics are more recalcitrant than carbohydrates, such as cellulose containing abundant O-alkyl groups^9^. Aliphatic compounds often accumulate in soil, thus contributing to increasingly stable SOC pools^10^,^11^. The alkyl/O-alkyl C of soil, which has been recommended as an index of the extent of decomposition^12^ and of the quality of SOC^13^,^14^, was also calculated in this study as an indicator of the chemical stability of SOC.

Chemically complex plant-derived compounds are known to be selectively preserved in the SOC fractions in terrestrial ecosystems^15^,^16^. Crow *et al.* (2009) showed that needle-derived aliphatic compounds and root-derived lignin were preferentially preserved in soils of coniferous forests, whereas root-derived aliphatic compounds were a source of SOC with greater stability than leaf-derived C in soils of deciduous forests, indicating that the dominant sources of SOC can differ substantially between forest types^8^. Mueller *et al.* (2013) reported that nearly 70% of the variation in individual soil lipid contents was explained by lipid contents in tree leaves and roots, whereas biological compositions, including bacteria and fungi, of soils had little impact on soil lipid contents^15^.

Although most SOC is initially derived from plant materials^4^, microbial-mediated decomposition and re-synthesis of plant input as the key processes shape stable soil C stocks^5^,^17^,^18^,^19^, giving rise to a theory that the plant litter has a rather fleeting influence on the quantity and composition of SOC^20^,^21^. This concept implies that plant litter chemistry may not be the main regulator controlling the amount and form of litter-derived C stabilized in soil^22^. Soil microbial biomass represents a significant source of SOC and contributes to the maintenance of soil organic matter (SOM) as a biochemical precursor^23^. The cell wall compounds, metabolites, and C use efficiencies all varied with microbial community compositions^24^. About 50% of bacterial biomass-derived C was found to remain in the soils^25^. The dynamics of root-associated fungi is also an important regulator of ecosystem C accumulation in boreal forests^26^. However, the input of easily degradable plant metabolites would destabilize SOC stocks by facilitating microbial co-metabolism of recalcitrant compounds^27^, or by enhancing microbial access to previously mineral-protected C compounds^28^, leading to a net loss of C from soil. In addition, You *et al.* (2016) pointed to differential controls on soil C density and mineralization among contrasting forest types and complex interplays among various biotic and environmental factors in regulating soil C dynamics^29^. The above complex processes highlight the existing disconnection between litter quality, variations in soil microbial community composition, and formation pathways of SOC.

Quantifying the effects of tree species on SOC is inevitably limited by soil background conditions in exiting forests, and the randomized block experiments with diverse tree species combinations may not be easily managed on the same site if long-term experimental maintenance and associated costs are taken into consideration. Thus, we conducted a manipulative leaf litter inputs field experiment under the same edaphic condition and site environment while excluding roots influence to examine the effects of leaf litter inputs from different tree species on SOC accumulation, and the associated underlying mechanisms. The first experiments on Detritus Input and Removal Treatments (DIRT) were established at Harvard Forest in temperate region in 1990 to address the gap between the above-ground versus below-ground litter input and their roles in determining soil C content and nutrient cycling in forest ecosystems^30^. There have been a few studies on the effects of plant litter inputs of aboveground and/or belowground on soil C cycling in tropical and subtropical forests^31^,^32^,^33^, in particular, under abundant water and heat conditions different from temperate climate^33^. However, data on the effects of different tree species’ litter inputs on chemical composition of SOC is lacking.

The objectives of this experiment were to examine the effects of leaf litter inputs from different tree species characterized with different chemical properties on soil microbial community composition and the chemical compositions of SOC in a *Pinus massoniana* plantation. Our hypotheses are as follows: (i) soil microbial community composition differ among the leaf litter inputs of four tree species; (ii) the soils that received different leaf litter inputs will exhibit the corresponding changes in chemical composition of SOC; (iii) the chemical composition of SOC is related to either leaf litter C chemical composition or soil microbial community composition, or their combinations and interactions.

## Results

### Leaf litter C chemical compositions

Significant differences were detected in the proportions of C chemical compositions (alkyl, O-alkyl, aromatic, and carbonyl C) and alkyl/O-alkyl C among the leaf litter samples of four tree species (*p* < 0.01). The proportions of alkyl C in *Castanopsis hystrix* and *Erythrophleum fordii* were significantly higher than in *P. massoniana* and *Cunninghamia lanceolate* (Table 1). The proportions of O-alkyl C in *P. massoniana* and *C. lanceolata* were higher than in *C. hystrix* and *E. fordii* (Table 1). Alkyl/O-alkyl C in *C. hystrix* and *E. fordii* were significantly higher than in *P. massoniana* and *C. lanceolate* (Table 1).

### Soil temperature and moisture, and microbial community composition

**Figure 1:**
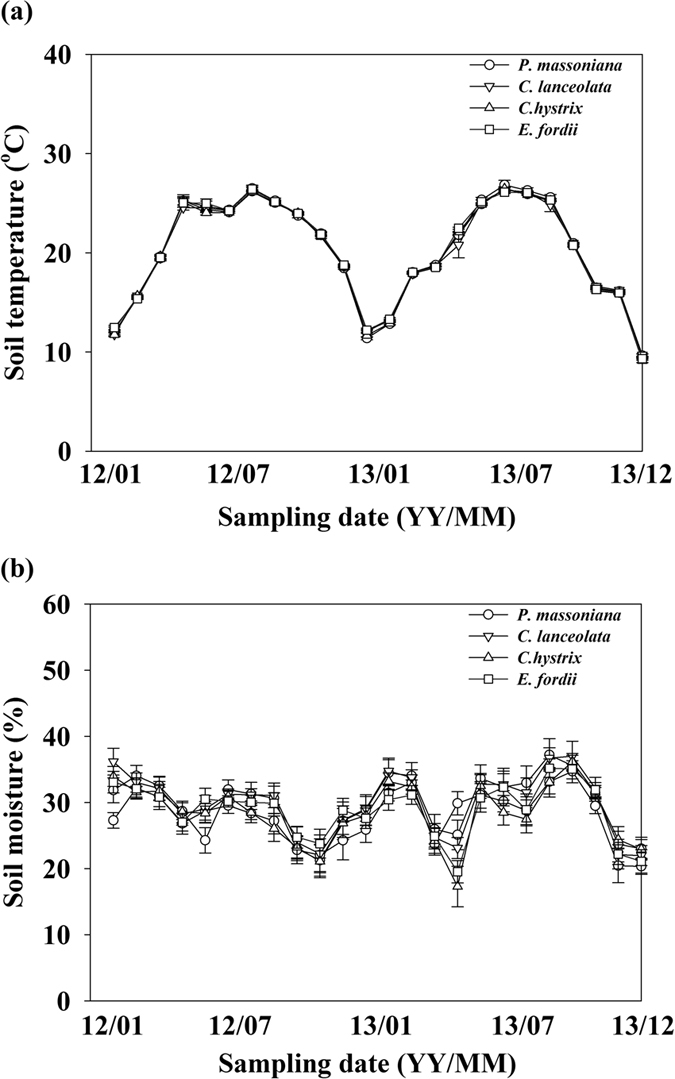
Soil temperature (a) and soil moisture (b) on a monthly basis from January 2012 to December 2013 in the different treatments of leaf litter inputs in a *Pinus massoniana* plantation in subtropical China. Bars show standard errors of the means (n = 6).

**Figure 2:**
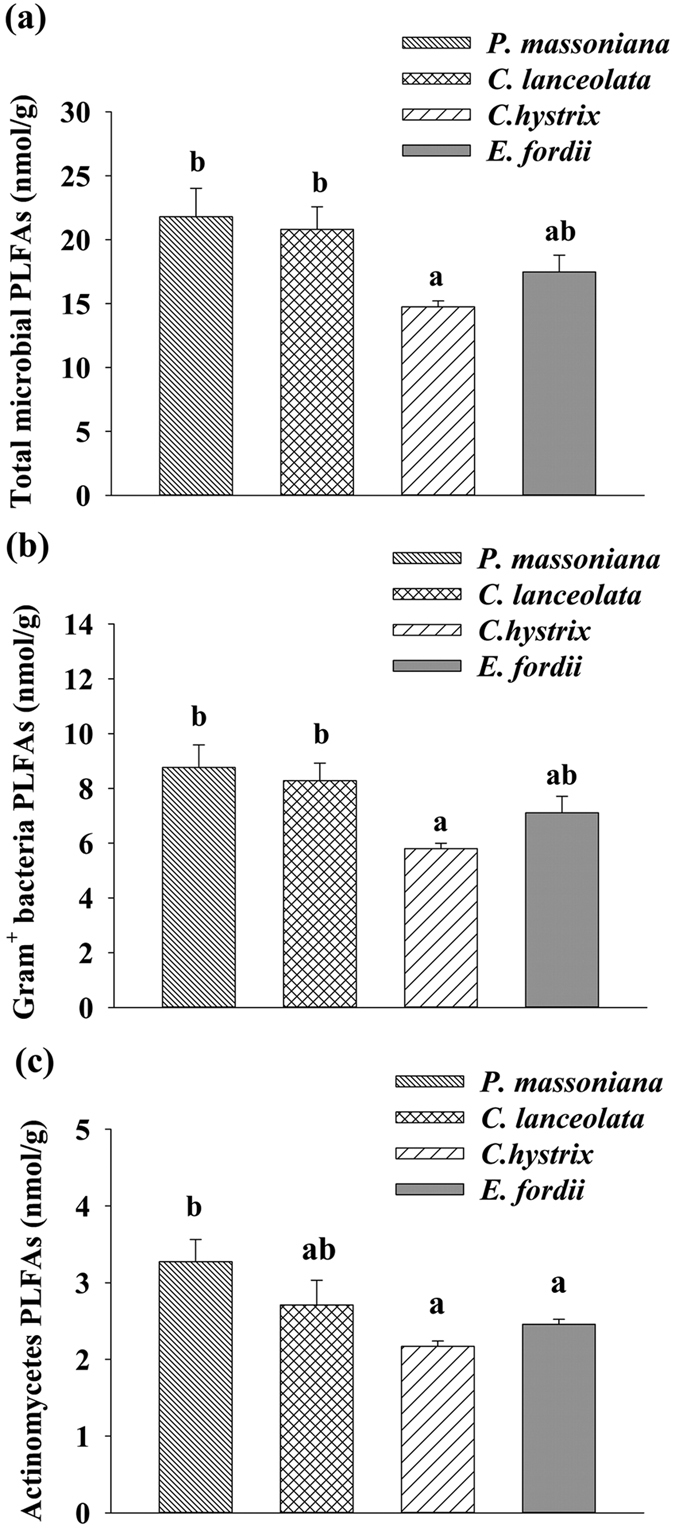
Soil phospholipid fatty acid (PLFA) amounts in the different treatments of leaf litter inputs sampled after 28 months of experiment, in a *Pinus massoniana* plantation in subtropical China. Bars show standard errors of the means (n = 3).

Neither soil temperature nor soil moisture significantly differed monthly from January 2012 to December 2013 among the four leaf litter input treatments (Fig. 1). In January 2014, the amounts of total microbial PLFAs, Gram-positive bacterial PLFAs, and actinomycic PLFAs were significantly different among the four treatments (*p* < 0.05). The amounts of total microbial PLFAs and Gram-positive bacterial PLFAs in the needle litter input treatments were significantly higher than in the *C. hystrix* leaf litter input treatments, but did not significantly differ with the *E. fordii* leaf litter input treatments (Fig. 2a,b). The amount of actinomycic PLFAs in the broadleaf litter input treatment was significantly lower than in the *P. massoniana* needle litter input treatment, but was not significantly different from that in the *C. lanceolata* needle litter input treatment (Fig. 2c). No significant variation occurred in the amounts of total bacterial PLFAs, Gram-negative bacterial PLFAs, fungal PLFAs, or in the fungi/bacteria (F/B) ratio among the four leaf litter input treatments (*p* > 0.05).

### SOC chemical compositions

**Figure 3:**
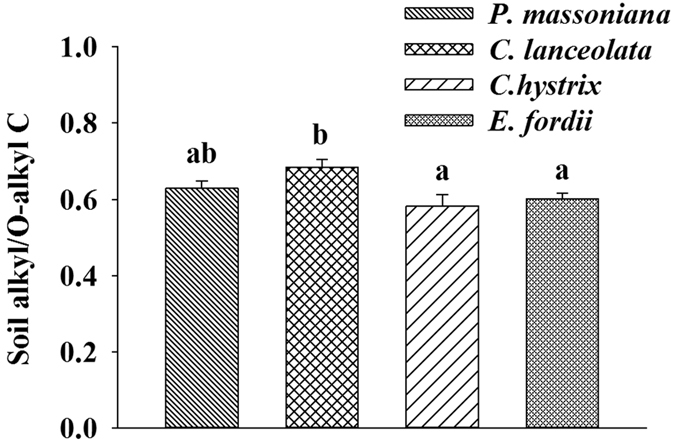
Soil alkyl/O-alkyl C in the different treatments of leaf litter inputs after 28 months of experiment in a *Pinus massoniana* plantation in subtropical China. Bars show standard errors of the means (n = 3).

After 14 months of the experiment, the proportions of alkyl, O-alkyl, aromatic, and carbonyl C in SOC, and soil alkyl/O-alkyl C did not differ significantly among the four leaf litter input treatments (Table 2; *p* > 0.05). However, after 28 months, soil alkyl/O-alkyl C significantly differed among the four leaf litter input treatments (*p* < 0.05); soil alkyl/O-alkyl C was significantly lower in the broadleaf litter input treatments than in the *C. lanceolata* needle litter input treatment, but was not significantly different with that in the *P. massoniana* needle litter input treatment (Fig. 3). However, the proportions of alkyl, O-alkyl, aromatic, and carbonyl C in SOC were not significantly different among the four leaf litter input treatments (*p* > 0.05).

### Relationships of soil alkyl/O-alkyl C with leaf litter C chemical compositions and soil microbial community composition

**Figure 4:**
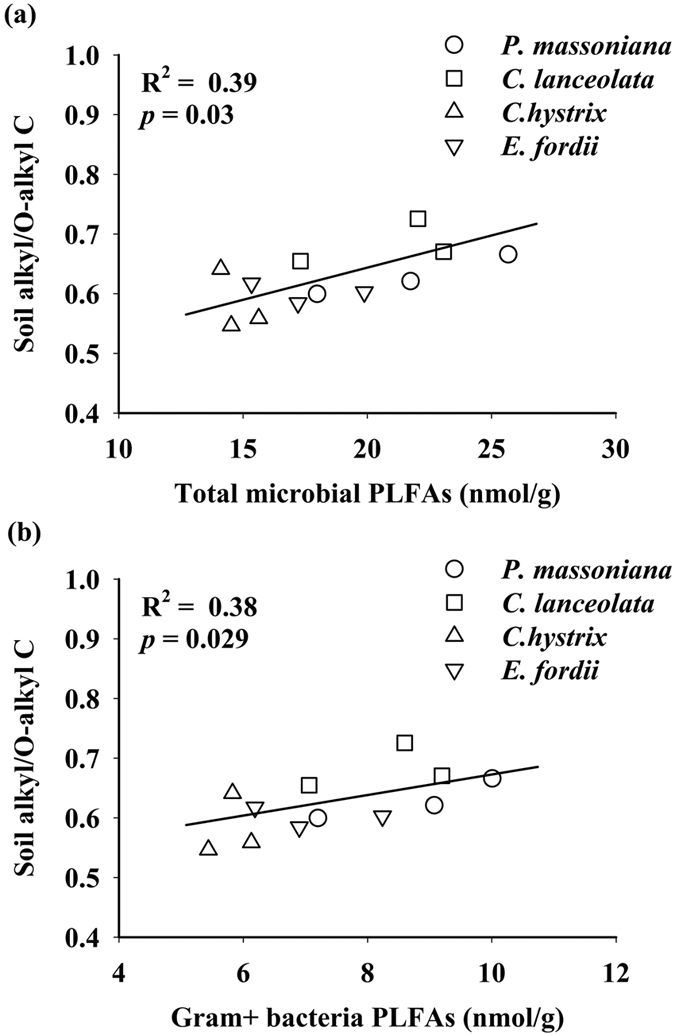
Relationships between soil alkyl/O-alkyl C and the amounts of total soil microbial and Gram-positive phospholipid fatty acids (PLFAs) across the leaf litter input treatments of the four tree species in a *Pinus massoniana* plantation in subtropical China.

Soil alkyl/O-alkyl C sampled in January 2014 was not correlated to the proportions of alkyl, O-alkyl, aromatic, and carbonyl C in leaf litter, nor was it to the leaf litter alkyl/O-alkyl C across the four leaf litter input treatments. However, the soil alkyl/O-alkyl C was significantly correlated to the amounts of total soil microbial PLFAs and Gram-positive bacterial PLFAs (Fig. 4a,b).

## Discussion

### Changes in soil microbial community composition induced by leaf litter inputs of different tree species

There were significant differences in the chemical compositions of leaf litter C among the four leaf litter types (Table 1). This helps understanding the role of chemical complexity of leaf litter C inputs in regulating the chemical composition of SOC. The chemical differences in leaf litter inputs can influence the chemical properties of both aggregated and non-aggregated SOM^34^. Changes in vegetation types and environmental conditions may affect the composition and function of decomposer community. In this study, we found that the amounts of total microbial, Gram-positive bacterial, and actinomycic PLFAs differed among the four leaf litter input treatments after 28 months of the experiment (Fig. 2), indicating shifts of soil microbial community composition with changes in litter type. Although very few studies have directly examined the effects of different tree species’ litter inputs on soil microbial community composition and consequently SOC dynamics, many studies, including the DIRT experiments and forest vegetation conversion studies^31^,^35^, showed the altered soil microbial community composition in response to the changes in plant C input. For example, Wang *et al.* (2013) reported that litter input and removal manipulation affected the soil microbial community composition in a central subtropical coniferous plantation^36^. We also found that the amounts of total PLFAs, fungal PLFAs, bacterial PLFAs, and Gram-positive and negative bacterial PLFAs significantly differed among four southern subtropical monospecific plantations^37^.

### Linkages of SOC chemical composition to leaf litter C chemical composition and soil microbial community composition

When the leaf litter input treatments persisted for 28 months, the chemical compositions of SOC in the 0–10 cm soil layer differed among the four leaf litter input treatments, as indicated by the significant differences in soil alkyl/O-alkyl C (Fig. 3). Furthermore, soil alkyl/O-alkyl C under the *C. lanceolata* needle litter input was significantly higher than in the broadleaf litter inputs (Fig. 3). The results suggest that the greater amount of relative stable C components accumulated in soils with treatments of the coniferous *C. lanceolata* leaf litter compared to the soils with treatments of the broadleaf litter of *C. hystrix* and *E. fordii*. Our results are supported by findings in a previous study that the chemical compositions of SOC differed among the four subtropical plantations, with a greater proportion of alkyl C and a lower proportion of O-alkyl C in SOC in *P. massoniana* plantation relative to the other three broadleaf plantations^38^. Also, the similar result was reported in a temperate forest that needle derived aliphatic compounds were preferentially preserved in soils of coniferous forests^39^.

The effects of vegetation type on SOC chemical composition could result from the diversity in chemical compositions of litter C and soil microbial necromass^40^,^41^. Plant litter contains various organic compounds including polysaccharides, aromatics, and aliphatics^8^. The quantity of litter and its chemical properties are the key factors influencing the formation of SOM in terrestrial ecosystems^42^. However, we did not find a clear relationship of the chemical compositions between SOC and leaf litter C in this study, suggesting that chemical composition of SOC could not be related to the chemical composition of leaf litter C; instead, some other alternative processes could be involved in the formation of soil C fractions. This result is in contrary to a previous study in temperate plantations that the concentrations of individual lipid in soil were very strongly correlated with their concentrations in leaves and roots^15^. The different results could be attributed to the different climate because fast leaf litter decomposition and fine roots turnover in tropical and subtropical forests. Our previous research has also showed no significant linkage between the chemical compositions of SOC and plant litter C in four subtropical plantations^37^.

The significant relationships between soil alkyl/O-alkyl C and the amounts of total microbial PLFAs, and Gram-positive bacterial PLFAs (Fig. 4) indicate that the chemical composition of SOC could be likely related to soil microbial community composition. It is consistent with the findings that litter loses mostly non-structural compounds, which are incorporated into microbial biomass at high rates, resulting in efficient SOM formation^43^. Soil microbes are considered as transformers of plant residue, and they use plant material as their C source, transforming it to CO_2_, intermediate metabolites, and soil microbial biomass^25^. The high abundance of submicrometer structures including fragments of hyphae, cells, cell wall fragments, and extracellular polysaccharides are related to microbes found in the soil^44^. In this study, only soil alkyl/O-alkyl C significantly differed among the four leaf litter input treatments. This is supported by the results of incubation experiment that microbial materials synthesized from glucose by soil microorganisms are mostly O-alkyl, alkyl, and carbonyl C, while phenolic and aromatic structures are only found in small amounts^45^. Furthermore, the positive relationship between the total soil microbial PLFAs and soil alkyl/O-alkyl C (Fig. 4a) suggests that the high abundance of soil bacteria and fungi could be linked to the high proportion of relatively stable aliphatic C in SOC. Webster *et al.* (2000) also reported that the soil alkyl/O-alkyl C increased in concomitant with the increased microbial activity during a 28-day incubation^46^. In addition, the positive relationship between Gram-positive bacterial PLFAs and soil alkyl/O-alkyl C (Fig. 4b) indicates that Gram-positive bacteria could be more related to the high proportion of stable aliphatic C in SOC than other soil microbial community compositions in this study. This is because that bacteria contains more alkyl C and less O-alkyl C than fungi^47^, and unique to Gram-positive bacteria is the presence of teichoic acids (containing lipid components) in the cell wall.

## Conclusion

Leaf litter inputs of the four subtropical tree species had the significant impact on the composition of soil microbial community, and consequently affected SOC chemical composition in the *P. massoniana* plantation. Organic C input from leaf litter materials did not directly contribute to the formation of SOC chemical fractions, whereas soil microbial community could be a main factor influencing the chemical composition of SOC. Our findings suggest that leaf litter input could induce changes in SOC chemical stability if a close-to-nature management is adopted in the subtropical region by substituting coniferous monospecific plantations with the native broadleaved tree species.

## Materials and Methods

### Site description

The experimental site is located at the Experimental Center for Tropical Forestry, the Chinese Academy of Forestry (22°05′N, 106°86′E), Pingxiang city, Guangxi Zhuang Autonomous Region, P. R. China. The climate is subtropical monsoon climate, with mean annual temperature of 22.3 °C and annual rainfall of 1400 mm falling mainly from April through September. The soils are classified as red soils based on Chinese soil classification, which is equivalent to Oxisol in USDA Soil Taxonomy^48^. Since 1950s, the majority of subtropical evergreen broadleaf forests have been harvested and subsequently replaced by Chinese fir (*C. lanceolata*) and masson pine (*P. massoniana*). In this study, a coniferous monospecific plantation dominated by *P. massoniana* at an elevation of 550 m was selected as experimental stand, which was established in 1983 after a clear-cut of a *C. lanceolata* plantation on the site. The mean diameter of trees measured at breast height (DBH), total tree height, and stem density of the *P. massoniana* plantation were 24.6 cm, 17.2 m, and 404 trees/ha, respectively^38^.

### Experimental design

In September 2011, a randomized complete block design with three blocks was established in the *P. massoniana* plantation. Each block was randomly designed to have four leaf litter input treatments. The area of each treatment is 2 m × 2 m. A total of 12 plots were included in the experiment. The four leaf litter input treatments of native tree species contained two coniferous tree species (*P. massoniana* and *C. lanceolata*) and two broadleaved tree species (*C. hystrix* and *E. fordii*). A 7–10 m buffer zone was in-between the plots to eliminate the interfering effect among the treatments. The averaged content of SOC across the 12 plots before the experiment was 37.6 ± 0.53 g/kg, with no significant difference in SOC among the four treatments (*p* > 0.05). Each of the plots was trenched to a depth of 1 m to minimize root growth entering the plots and the outside edges of the trenches were lined with thick plastic sheets and backfilled with soil. There were no trees and shrubs inside the plots, and litter detritus and understory were removed from the plots before the experiment. Natural litterfall from the adjacent trees was blocked by nylon mesh spread at a height of 1.6 m above each plot. The plots were monitored and kept free from plants throughout the experiment. The *P. massoniana* needle litter was collected in the same plantation site, and the *C. lanceolata*, *C. hystrix*, and *E. fordii* leaf litter were collected in the three adjacent monospecific plantation sites, respectively, with the similar topography, soil texture, stand age, and site history. In each of the four plantation sites, 20 litter traps (each 1 m × 1 m) with a mesh size of 1 mm were randomly installed at a height of 1 m above the ground. This work was conducted based on the Forestry Standards “Observation Methodology for Long-term Forest Ecosystem Research” of the People’s Republic of China (LY/T 1952–2011). Leaf litter of each monospecific plantation was monthly collected and mixed together. One fifth of the collected leaf litterfall was oven dried at 65 °C for a constant weight and then weighed for calculating moisture content. Another one fifth of the leaf litterfall was reserved for chemical analysis. The remaining leaf litter inputs was monthly added on the soil surface in each of experimental plots at the same mass of dry weight, which was defined by the minimum value of leaf litter mass of dry weight among the four monospecific plantations. From September 2011 to December 2013, 241.5 g m^−2^ yr^−1^ of litter mass of dry weight was added to the 12 plots.

### Samplings and measurements

The reserved samples of leaf litterfall from each of the four monospecific plantations which were collected monthly from January 2012 to December 2013, were pooled together, respectively, for analysis of major C chemical compositions (i.e., alkyl, O-alkyl, aromatic, and carbonyl C) by using solid-state ^13^C cross polarization with magic angle spinning nuclear magnetic resonance (CPMAS NMR)^49^,^50^. The ^13^C CPMAS NMR spectra were obtained at a frequency of 100.64 MHz on a Bruker AVANCE III 400 spectrometer (Bruker, Karlsruhe, Germany). Samples were packed in a ZrO_2_ rotor (OD = 7 mm) and spun at 5 kHz at the magic angle. Single contact time of 1 ms was applied with an acquisition time of 42 ms, and a recycle delay of 1 s. Transients (20,000) were collected for all samples and a Lorentzian line broadening function of 50 Hz was applied to all spectra. Chemical shift values were referenced externally to glycine at 176.03 ppm, which is equivalent to tetramethylsilane at 0 ppm. The ^13^C CPMAS NMR spectra were divided into four chemical regions that were assigned to specific organic C functional groups^4^,^51^: 0–45 ppm, alkyl C (lipids, cutin, and suberin): 0–45 ppm, alkyl C (lipids, cutin, and suberin); 45–110 ppm, O-alkyl C (carbohydrates, cellulose, hemicelluloses, and methoxyl C); 110–160 ppm, aromatic C (lignin, tannin, olefins, and aromatic compounds); and 160–220 ppm, carbonyl C (carboxylic acid, amide, and ketone groups). The corresponding areas under the curve of the above four regions were quantified by integration.

Mineral soil samples were collected in November 2012 and January 2014, respectively, i.e. 14 and 28 months after the start of the experiment, to determine the effects of different characterized leaf litter inputs on the chemical composition of SOC. Four 5-cm-diameter soil cores were randomly taken to a depth of 10 cm in each plot. The four cores of each plot were combined and sieved through a 2-mm sieve to carefully remove plant material, roots, and gravel to minimize the influence of the plant residues on both chemical and microbial analyses. Soil samples were ground for analyzing for major organic C chemical compositions. The soil samples were pretreated with 10% (v/v) hydrofluoric acid solution prior to solid-state ^13^C CPMAS NMR spectra analysis, to remove a substantial amount of Fe^3+^ and Mn^2+^ from soils and to concentrate SOC for improving the signal/noise ratio of NMR^49^.

The fresh soil samples in January 2014 were analyzed for phospholipid fatty acids (PLFAs) following Bossio and Scow^52^. The abundance of individual fatty acids was determined as nmol per g of dry soil using standard nomenclature^53^. In this study, the content of each PLFA was calculated based on the contents of the 19:0 internal standards. Bacteria were identified by the following PLFAs: i14:0, i15:0, a15:0, 15:0, i16:0, a17:0, i17:0, 15:0 3OH, 16:1 2OH, cy17:0, 17:0, 16:1ω7c, and 18:1ω7c^54^,^55^. We calculated the sum of i14:0, i15:0, a15:0, 15:0, i16:0, a17:0, and i17:0 as Gram-positive bacteria^56^,^57^ and the sum of 15:0 3OH, 16:1 2OH, cy17:0, 17:0, 16:1ω7c, and 18:1ω7c as Gram-negative bacteria^55^. The content of PLFAs methyl branched fatty acids (Me) was calculated as a group of actinomycetes^58^. Fungi were identified by the PLFAs 18:2ω6, 9c, and 18:1ω9c^58^,^59^. All of the above PLFAs were used to represent the total PLFAs of the soil microbial community.

Soil temperature and moisture at 5 cm below the soil surface were monitored in each plot. Soil temperature was measured using a digital thermometer (Harvesting Science and Technology Co., Ltd, BJ, China). Volumetric soil moisture (% v/v) was measured simultaneously using Mpkit-B (NTZT Inc., Nantong, JS, China), which consisted of four amplitude domain reflectometry (ADR) moisture probes (MP406). The measurements of soil temperature and soil moisture were made twice a month from January 2012 to December 2013. The 2-monthly measurements of soil temperature and soil moisture in each plot were averaged for each month.

### Statistical analysis

One-way analysis of variance was used to examine leaf litter input effects of the four tree species on the proportions of organic C chemical compositions (alkyl, O-alkyl, aromatic, and carbonyl C) in SOC, soil alkyl/O-alkyl C, and the amounts of soil microbial PLFAs. The differences in the proportions of C chemical compositions (alkyl, O-alkyl, aromatic, and carbonyl C) in leaf litter C, and leaf litter alkyl/O-alkyl C among the four tree species were also analyzed using one-way analysis of variance. The statistically significant level was set at *p* < 0.05. Multiple comparisons of mean among the four treatments were subjected to Duncan’s test. Soil alkyl/O-alkyl C was related to leaf litter C chemical compositions and the amounts of soil microbial PLFAs across the four treatments using bivariate linear regressions. All analyses were performed using SPSS 19.0 for Windows.

## Additional Information

**How to cite this article**: Wang, H. *et al.* Differential effects of conifer and broadleaf litter inputs on soil organic carbon chemical composition through altered soil microbial community composition. *Sci. Rep.* **6**, 27097; doi: 10.1038/srep27097 (2016).

## Figures and Tables

**Table 1:** Leaf litter C chemical compositions of the four tree species in subtropical China (means with SE in brackets).

Leaf litter C chemical composition	*P. massoniana*	*C. lanceolata*	*C.hystrix*	*E. fordii*
Proportion of alkyl C (%)	15.2 (0.5) a	15.4 (0.4) a	16.7 (0.2) b	19.3 (0.1) c
Proportion of O-alkyl C (%)	60.1 (0.6) c	58.9 (0.7) c	56.2 (0.1) b	53.0 (0.1) a
Proportion of aromatic C (%)	18.2 (0.5) a	18.9 (0.1) a	19.8 (0.2) b	18.3 (0.1) a
Proportion of carbonyl C (%)	6.5 (0.5) a	6.8 (0.2) a	7.4 (0.1) a	9.5 (0.1) b
Alkyl/O-alkyl C	0.25 (0.01) a	0.26 (0.01) a	0.30 (0.01) b	0.36 (0.01) c

Different lowercase letters indicate significant differences among different tree species at *p* < 0.05 (n = 3).

**Table 2:** Soil organic C chemical compositions of different leaf litter input treatments after 14 months in a *Pinus massoniana* plantation in subtropical China (means with SE in brackets).

Soil organic C chemical composition	*P. massoniana*	*C. lanceolata*	*C.hystrix*	*E. fordii*
Proportion of alkyl C (%)	25.0 (0.2) a	24.4 (0.3) a	23.1 (1.6) a	26.2 (1.0) a
Proportion of O-alkyl C (%)	41.7 (1.2) a	40.6 (1.4) a	40.3 (1.7) a	42.1 (2.7) a
Proportion of aromatic C (%)	23.9 (0.2) a	24.4 (0.9) a	26.0 (2.0) a	23.1 (1.2) a
Proportion of carbonyl C (%)	9.4 (1.3) a	10.5 (0.9) a	10.5 (1.5) a	8.5 (1.1) a
Alkyl/O-alkyl C	0.60 (0.01) a	0.60 (0.03) a	0.57 (0.02) a	0.63 (0.07) a

Different lowercase letters indicate significant differences among different tree species at *p* < 0.05 (n = 3).
